# EEG Characteristics in COVID-19 Survivors and Non-survivors With Seizures and Encephalopathy

**DOI:** 10.7759/cureus.18476

**Published:** 2021-10-04

**Authors:** Bhanu Gogia, Neeharika Thottempudi, Yousaf Ajam, Ayush Singh, Tamer Ghanayem, Alok Dabi, Xiang Fang, Todd Masel, Prashant Rai

**Affiliations:** 1 Vascular Neurology/Neurology, Beth Israel Deaconess Medical Center, Harvard Medical School, Boston, USA; 2 Neurology, University of Texas Medical Branch at Galveston, Galveston, USA; 3 Neurology, Weill Cornell Medical Center, New York, USA; 4 Neurology, Emory University, Atlanta, USA

**Keywords:** covid-19 and eeg findings, encephalopathy and covid-19, covid-19 and seizures, status epilepticus in covid-19, neurological manifestations in covid-19

## Abstract

The objective of this study is to report EEG findings in both COVID-19 survivors and non-survivors who underwent EEG either due to seizure or encephalopathy. Out of total 1468 COVID-19-positive patients, 19 patients underwent EEG. Eight out of 19 patients had a history of seizure disorder and in the remaining 11 with no prior history of seizures, four had a clinical seizure during their hospital stay. Only one had new-onset complex focal status epilepticus on EEG. Amongst the survivors (13/19), the most common EEG findings were normal followed by mild diffuse slowing. Amongst the non-survivors (6/19), the most common EEG finding was moderate to severe slowing in 50% of the patients. It can be deduced that COVID-19 infection does not increase the propensity of epileptiform discharges on EEG. There is perhaps a trend towards increased risk of new-onset status epilepticus in patients with encephalopathy and focal lesions.

## Introduction

Seizures have been reported as one of the presenting neurological symptoms of COVID-19 infection [1]. Some causes of provoked seizure, either new onset or a breakthrough seizure in a patient with known epilepsy, are structural brain lesions, systemic or meningo-encephalitic infection, electrolyte abnormalities, missing dosages of anti-epileptic drugs (AEDs), and disturbances in sleeping patterns [2]. With increasing literature on neurological associations of COVID-19 infection, both patients with new-onset seizure and increase in seizure frequency have been reported [3,4]. In relationship to COVID-19, some of the possible mechanisms reported to be responsible for seizures include neuroinvasion, hypoxia, metabolic derangements, organ failure, and therapeutic drugs [5]. Stress is also a well-known trigger for both epileptic and non-epileptic seizures [6]. COVID-19 has caused immense stress and anxiety amongst people worldwide (16-28%) [7-9]. In addition, the shift in paradigm to telehealth has also impacted overall health-care delivery. 

While there is budding literature on increased frequency of clinical seizures during the pandemic, the knowledge about EEG findings in patients with COVID-19 infection is thus far limited. The current evidence does not suggest any increased risk of infection amongst people with seizure disorder [10]. There are studies reporting EEG findings such as background slowing and generalized epileptiform discharges with triphasic morphology in 3/8 patients, and lateralized periodic discharges consisting of symmetric slow monomorphic biphasic delta waves and focal seizure in 1/8 patient with prior history of seizures [11-16]. The aim of this study is to report EEG findings in COVID-19 patients at our institution who underwent EEG either due to seizure or encephalopathy.

## Materials and methods

This is a single-center retrospective study. After obtaining approval from institution review of board (IRB)# 20-0142.015, the authors (BG, AD, XF and PKR) prepared a questionnaire in the Microsoft forms software. The questionnaire contained 74 questions pertaining to demographics including age, sex, ethnicity, medical history, social history, presenting symptoms, hospital course, and disposition. The charts of the patients with COVID-19 who were admitted to the hospital from February to August 2020 and underwent EEG were reviewed in EPIC (developed in Verona, Wisconsin, USA). All data were collected and entered into the Microsoft forms (by YA, AS, NT and TG). All EEGs were recorded in 27 channels, and were either short duration 20-30 minutes or long-term EEG. All EEGs were interpreted by a board-certified neurophysiologist (PKR and TM). The data were then tabulated in a Microsoft Excel sheet, and the data analysis was done in the Microsoft Excel sheet using simple calculations without the use of any statistical methods. 

## Results

Patient characteristics

Of the total 1468 patients who were admitted at the hospital between February 1st 2020 and August 19th 2020, only 19 patients underwent EEG. The indications of EEG based on the questionnaire were altered mental status at presentation, concern for non-convulsive status epilepticus (NCSE), clinically noted seizure or gaze deviation. Of the 19 patients studied, 14 were males and five were females, with age ranging from 20 to 88 years (average: 60.73 years), out of which six were Caucasians, six African Americans, six Hispanics and one Asian. 

Onset of seizure and reason for EEG 

Eight out of 19 patients had a history of seizure disorder. Of the remaining 11 patients with no prior history of seizures, four had a clinical seizure during their hospital stay. Neurology was consulted in 18/19 patients, with the reason for consult as clinical seizure in 11/18 and altered mental status in 7/18. In those patients where a clinical seizure was observed, the semiology was described as tonic-clonic generalized convulsive activity, except for one patient who had focal onset seizure with secondary generalization which is described as left-sided shaking with left gaze deviation. 

AED levels and self-reported stress

AED levels were checked in 5/8 patients with known history of seizure disorder because five had reported clinical seizures during hospitalization. Three of five patients had subtherapeutic AED levels, and 2/5 had self-reported stress due to COVID-19.

Electrographic findings 

**Figure 1:**
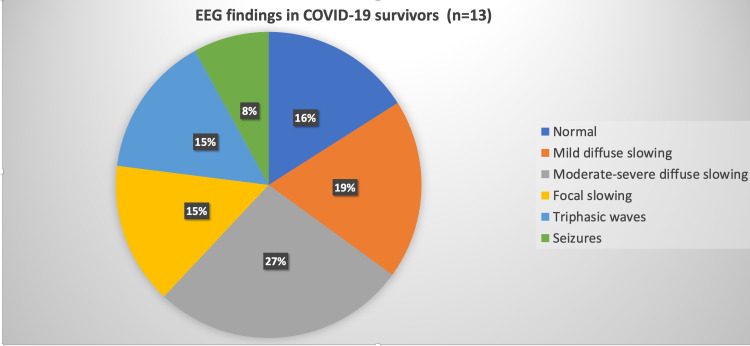
Graphical representation of EEG characteristics in COVID-19 survivors

**Figure 2:**
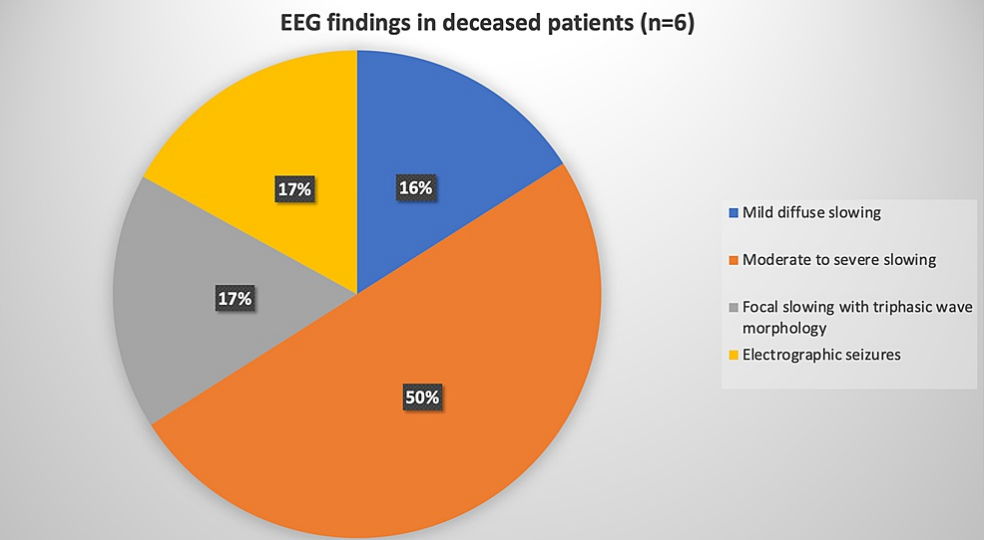
Graphical representation of EEG characteristics in COVID-19 non-survivors

While EEG was obtained, 7/19 patients were intubated and sedated due to their respiratory decompensation. Of these seven patients, one had electrographic seizure activity and focal onset status epilepticus. Of those who were on room air (n=12), only 1/12 had an electrographic seizure. The EEG findings are demonstrated in Figures 1, 2 and are described further in Table 1.

**Table 1:** Overall EEG characteristics along with positive neuroimaging findings MCA - middle cerebral artery, ICA - internal carotid artery

EEG findings	Number of patients	Neuroimaging findings
1. Normal	4	
2. Mild diffuse slowing		
2a. With triphasic morphology	1	
2b. Without triphasic morphology	3	
3. Moderate-severe slowing		
3a. With triphasic morphology	1	
3b. Without triphasic morphology	3	
4. Focal slowing		
4a. Right fronto-temporal slowing, moderate diffuse slowing	1	Right temporal occipital subdural hemorrhage
4b. Right hemispheric slowing with moderate diffuse slowing with triphasic morphology	1	Subacute right MCA infarct with non-occlusive thrombus in right intracranial ICA
4c. Left temporal slowing with mild-moderate diffuse slowing	1	Acute left thalamic hemorrhage
4d. Bitemporal slowing	1	
4e. Bilateral independent fronto-temporal epileptiform discharges, moderate-severe slowing	2	
5. Electrographic seizures/status epilepticus	1	No imaging obtained

Neuroimaging findings

Neuroimaging (non-contrast CT head and/or MR brain without contrast) was obtained in 16/19 patients. From the 16 patients who underwent neuroimaging, 11 had no acute findings, two had acute intraparenchymal hemorrhage, one had acute subdural hematoma, one had sulcal effacement and diffuse cerebral edema concerning for hypoxic/anoxic brain injury, and one had right middle cerebral artery (MCA) acute/subacute infarct.

Patient outcomes and EEG findings

Death was reported in 6/19 (31.5%) patients. Five of these six deceased patients were in the intensive care unit and four of them were intubated. Three of these patients had altered sensorium for which EEG was ordered to evaluate for NCSE, while two had observed motor seizure and one had a witnessed clinical seizure at an outside facility. Of all deceased, one patient had a prior history of seizure disorder. The EEG findings in the COVID-19 patients based on their outcomes are demonstrated in Figures 1, 2. 

## Discussion

**Figure 3:**
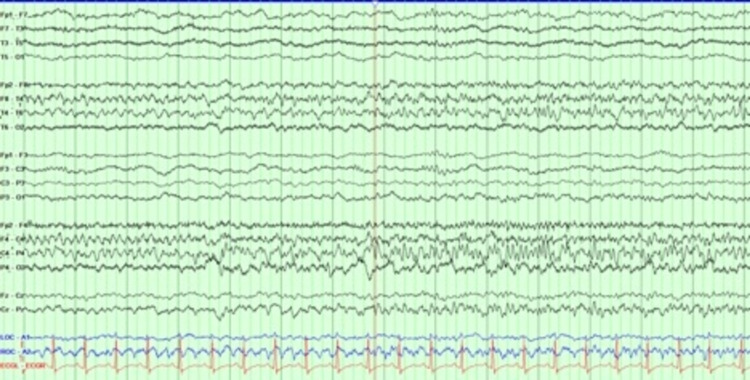
Double banana montage of EEG showing focal seizure originating from right temporal region

**Figure 4:**
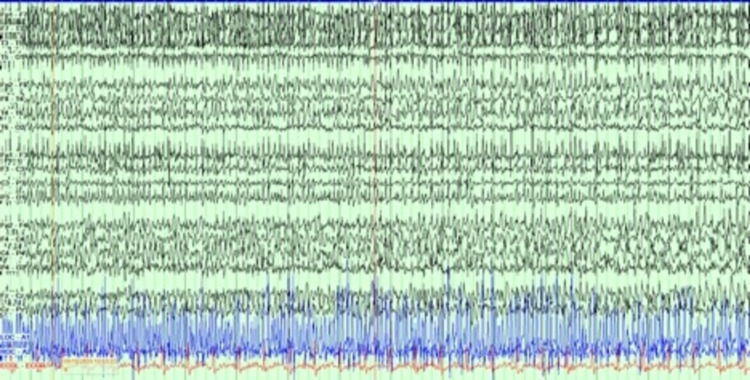
Double banana montage of EEG showing seizure spread to the right hemisphere with secondary generalization

There have been increasing reports of new-onset seizures as well as increased frequency of breakthrough seizures in patients with COVID-19 [3-5,10]. While the underlying pathology is still unclear, many theories have been proposed, including neurological, systemic factors, and stress, which can directly or indirectly trigger seizures in patients with COVID-19 [6-9]. It is known that patients with no prior history of epilepsy who present with new NCSE carry a poor prognosis compared to those with known history of seizures [17]. Unlike the findings reported by Lu et al. [10], in the group of patients presented here, there was one patient (patient #19) with new-onset focal status epilepticus with secondary generalization. The patient did have positive blood culture growing *Escherichia coli* but otherwise no identified electrolyte abnormalities that would be thought to trigger a seizure. The EEG patterns of this patient are shown and described in Figures 3, 4. Unfortunately, head imaging was not obtained as the patient was hemodynamically unstable and passed away before obtaining imaging. It is a possibility that there was a focal lesion to explain the focal onset of status epilepticus. Another patient (patient #10) was transferred from an outside facility with concerns of status epilepticus. The patient arrived intubated and sedated, and had no electrographic seizures, but CT head showed a subacute right MCA infarct. Similar to Chen et al. [13], our cohort also had two patients with new-onset status epilepticus, one without known provoking factors, and the other developed seizure secondary to infarct. However, it cannot be said if the infarct developed secondary to COVID-19 infection or was a co-incidental finding. 

In the individuals with prior history of epilepsy (8/19), only five (62.5%) had a clinical seizure similar to their typical episode. Although multiple factors may have contributed to the seizures including subtherapeutic AED levels in three patients, and self-reported stress due to COVID-19 in two patients, none of the patients in this study had any electrolyte abnormalities. 

Although other studies have reported a unique EEG finding consisting of monomorphic, bipolar diphasic delta waves with slow projections [14,15] or epileptiform discharges with frontal sharp waves [16], we could not duplicate these findings in our cohort. Three patients had triphasic morphology discharges with diffuse slowing on EEG. Triphasic waves are a common EEG finding seen with toxic-metabolic encephalopathy [18], and it is interesting to note that in all three patients, the reason for neurology evaluation was altered mental status, but only one had an identified toxic-metabolic cause with deranged liver and renal functions. 

Amongst the deceased patients (n=6), the most common EEG finding was moderate-to-severe slowing in 50% of the patients, followed by mild diffuse slowing in 33.3 % (n=1), focal slowing with triphasic morphology discharges in 33.3% (n=1), and electrographic seizure in 33.3%. Similar to Pilato et al. [12], in our cohort also, generalized moderate-to-severe slowing was the most common finding in patients with fatal outcome. Such degree of generalized diffuse slowing on EEG is suggestive of severe encephalopathy, which in this cohort might be alluding to the severity of the COVID-19 infection. 

Limitation of this study

It is a retrospective descriptive analysis of a relatively small cohort and no controls to match. In the patients with history of epilepsy and those who had seizures during hospital stay, a long-term EEG would have been more useful to rule out seizures and NCSE. 

## Conclusions

From this data, no specific abnormal pattern was seen on EEG in COVID-19 patients but more than 50% of the deceased patients had generalized diffuse severe slowing indicating a global process. There is perhaps a trend towards increased risk of new-onset status epilepticus in patients with encephalopathy and focal lesions. These findings may assist physicians in leaning towards a more aggressive AED regimen in management of COVID patients with seizures and focal lesions. Further studies are needed with larger sample size, longer duration of EEG monitoring as well as EEG in post-recovery phase in patients with new-onset seizures.
